# Memorial Edition

**Published:** 1893-06

**Authors:** 


MEMORIAL EDITION.—As usual with the beginning of a new volume the JOURNAL will issue for July 1500 copies of 100 pages. The leading article in the July number will be the report of the Newburg Case by Professor D. R. Wallace, mentioned elsewhere. Advertisers should take advantage of this extra issue and place their orders for space without delay. Will make speecial rates for that one edition.

